# Not taking sick leave for gynecologic cancer treatment is negatively associated with returning to the same workplace

**DOI:** 10.1093/jjco/hyad159

**Published:** 2023-11-23

**Authors:** Keiichiro Nakamura, Hirofumi Matsuoka, Kotaro Kubo, Shinsuke Shirakawa, Naoyuki Ida, Junko Haraga, Chikako Ogawa, Kazuhiro Okamoto, Shoji Nagao, Hisashi Masuyama

**Affiliations:** Department of Obstetrics and Gynecology, Okayama University Graduate School of Medicine, Dentistry and Pharmaceutical Sciences, Okayama, Japan; Department of Obstetrics and Gynecology, Okayama University Graduate School of Medicine, Dentistry and Pharmaceutical Sciences, Okayama, Japan; Department of Obstetrics and Gynecology, Okayama University Graduate School of Medicine, Dentistry and Pharmaceutical Sciences, Okayama, Japan; Department of Obstetrics and Gynecology, Okayama University Graduate School of Medicine, Dentistry and Pharmaceutical Sciences, Okayama, Japan; Department of Obstetrics and Gynecology, Okayama University Graduate School of Medicine, Dentistry and Pharmaceutical Sciences, Okayama, Japan; Department of Obstetrics and Gynecology, Okayama University Graduate School of Medicine, Dentistry and Pharmaceutical Sciences, Okayama, Japan; Department of Obstetrics and Gynecology, Okayama University Graduate School of Medicine, Dentistry and Pharmaceutical Sciences, Okayama, Japan; Department of Obstetrics and Gynecology, Okayama University Graduate School of Medicine, Dentistry and Pharmaceutical Sciences, Okayama, Japan; Department of Obstetrics and Gynecology, Okayama University Graduate School of Medicine, Dentistry and Pharmaceutical Sciences, Okayama, Japan; Department of Obstetrics and Gynecology, Okayama University Graduate School of Medicine, Dentistry and Pharmaceutical Sciences, Okayama, Japan

**Keywords:** returning to the same workplace, gynecologic neoplasms, sick leave

## Abstract

**Background:**

Gynecologic cancers are one of the most common types of malignancies in working-age women. We aimed to determine the factors that impede women from returning to the same workplace after treatment for such cancers.

**Methods:**

A questionnaire-based survey was conducted on 194 women who underwent treatment for gynecologic cancer at the Okayama University (≥1 year after cancer treatment and <65 years of age). We performed a logistic regression analysis to determine the relationship between returning to the same workplace and not taking sick leave.

**Results:**

The median age at diagnosis was 49.0 years, and the median time from cancer treatment to questionnaire completion was 3.8 years. Not returning to the same workplace was positively associated with not being regularly employed (*P* = 0.018), short work time per day (*P* = 0.023), low personal income (*P* = 0.004), not taking sick leave (*P* < 0.001), advanced cancer stage (*P* = 0.018) and long treatment time (*P* = 0.032). Interestingly, not taking sick leave was strongly associated with not returning to the same workplace in the multivariable analysis (*P* < 0.001).

**Conclusions:**

Not taking sick leave likely was negatively associated with returning to the same workplace after the treatment for gynecologic cancer. Therefore, we suggest that steps be taken to formally introduce a sick leave system over and above the paid leave system in Japan.

## Introduction

Gynecologic cancers, including cervical, endometrial and ovarian cancers, are the fifth most common types of cancer in Japanese women (1). Most patients with cervical cancer are diagnosed in their 20s–40s (2), and those with endometrial or ovarian cancer are typically diagnosed in their 40s and 50s (3,4). Gynecologic cancers are clearly common among working-age women.

In one systematic review, women with cancer of the reproductive organs were 1.28 times more likely to be unemployed than healthy control participants (5). Returning to the same workplace rates range from 53.8 to 95.3% for all cancers and from 42.9 to 95.2% for female genital cancer in Japan (6). According to our 2015 survey results, 71.3% of patients returning to the same workplace and 12.6% of patients changed jobs (7). The Japanese government amended the Cancer Control Act in 2016 to improve the returning to the same workplace rate after cancer treatment and published a national guideline for support of the work life of such individuals (8).

Returning to the same workplace may help patients overcome the negative impacts of disease treatments, improve patients’ financial situation and reduce the economic burden of cancer on society (9,10). Returning to the same workplace is very important not only for the individual concerned but also for their families, employers and society. No reports have been published in which the situation before and after the revision of the Cancer Control Act was compared; therefore, we conducted a questionnaire survey in our department with the aim of investigating the current situation regarding returning to the same workplace of women undergoing treatment for gynecologic cancers.

## Patients and methods

### Study population

We distributed questionnaires to 253 women with gynecologic cancer (surviving ≥1 year after cancer treatment and aged <65 years), who visited Okayama University between 5 January and 27 April 2023. At diagnosis, 194 patients were employed and 59 were unemployed. Patients with recurrent or relapsed disease were excluded from the study. All patients were informed of the survey by their consultant doctors and provided written informed consent to participate in this study. Responses were voluntary, and completed questionnaires were collected using an in-hospital collection box. Questionnaires were completed by almost all the patients (99.2%) who consented to participate. The study protocol was approved by the Institutional Review Board of Okayama University Hospital (no: 2212-034). The dataset comprised responses from 194 women who had been employed and working at the time of their cancer diagnosis. Data on marriage status, number of children, cancer site, cancer stage and cancer treatment were extracted from the participants’ medical records. The questionnaire used in this study was based on the contents of the 2015 questionnaire we previously created (7). It contained questions on participants’ employment pattern, workdays per week, work hours per day, number of people in the workplace, personal income, household income and taking sick leave. The sick leave taken covered a wide period before and after treatment (including for the initial treatment and post-treatment). However, this study did not examine the content of sick leave in detail.

### Study variables

Employment status at the time of diagnosis was divided into the following four categories: (i) self-employed, (ii) public servant (regularly employed), (iii) regularly employed (permanent employment) and (iv) non-regularly employed (part-time, temporary, contract-based and dispatched workers). We also investigated age, marital status, having children, cancer site, cancer stage, cancer treatment duration, employment pattern, working days per week, working hours per day, number of people at the workplace, personal income, household income, taking sick leave (paid vacation or other types of leave), return to the same workplace and periods of cancer treatment. In this study, we investigated the returning to the same workplace status in the immediate post-treatment period. Patients who quit or changed jobs after returning to the same workplace were defined as returning to the same workplace for the purposes of this study.

### Statistical analysis

SPSS Statistics software, version 26.0 (IBM Corp., Armonk, NY, USA) was used to perform statistical analyses. Between-group differences were assessed using the Wilcoxon rank-sum test, *χ*^2^ test and Steel test, as appropriate. We examined it with the same cut-off as the previous questionnaire in workdays per week, work hours per day, number of people in the workplace, personal income and household income (7). We used univariate and multivariable logistic regression analyses to investigate returning to the same workplace and not taking sick leave. A *P*-value of <0.05 was considered to be statistically significant.

## Results

All 194 gynecologic cancer survivors completed the questionnaire during their outpatient visits. The median age at cancer diagnosis was 49.0 years (range: 29–64 years), and the median time from cancer treatment to questionnaire completion was 3.8 years. Regarding the employment status at the time of cancer diagnosis, 20 patients (10.3%) were self-employed, 11 (5.7%) were public servants, 87 (44.8%) were regularly employed and 76 (39.2%) were non-regularly employed. Patient characteristics are summarized in Table 1.

**Table 1:** Patient characteristics at the time of cancer diagnosis

Age at diagnosis (years)	Median: 49.0, range: 29–64	
	Numbers	(%)
Married		
Yes	153	78.9
No	41	21.1
Children		
Yes	143	73.7
No	51	26.3
Cancer site		
Cervical cancer	79	40.7
Endometrial cancer	86	44.3
Ovarian cancer	23	11.9
Other cancers	6	3.1
Stage		
Early	156	80.4
Advanced	38	19.6
Treatment		
Surgery	77	39.7
Surgery + chemotherapy (three courses)	16	8.2
Surgery + chemotherapy (six to nine courses)	29	14.9
Radiation (including CCRT)	27	13.9
Surgery + radiation (including CCRT)	10	5.2
Employment pattern		
Self-employed	20	10.3
Public servant	11	5.7
Regularly employed	87	44.8
Non-regularly employed	76	39.2
Work days per week		
≤3	14	7.2
4	18	9.3
5	135	69.6
≥6	23	11.9
Work hours per day		
≤5	48	24.7
6–8	78	40.2
>8	68	35.1
Workplace number of people		
≤5	51	26.3
6–10	34	17.5
11–20	33	17
21–30	18	9.3
31–50	23	11.9
>50	35	18
Personal income (10 000 yen)		
<103	50	25.8
103–149	42	21.6
150–299	54	27.8
300–499	37	19.1
≥500	11	5.7
Household income (10 000 yen)		
<300	43	22.2
300–499	49	25.3
500–699	46	23.7
700–999	43	22.2
1000–1499	12	6.2
≥1500	1	0.5
Sick leave		
Taking sick leave	142	73.2
Not taking sick leave	52	26.8

Abbreviation: CCRT, concurrent chemoradiotherapy.

**Figure 1:**
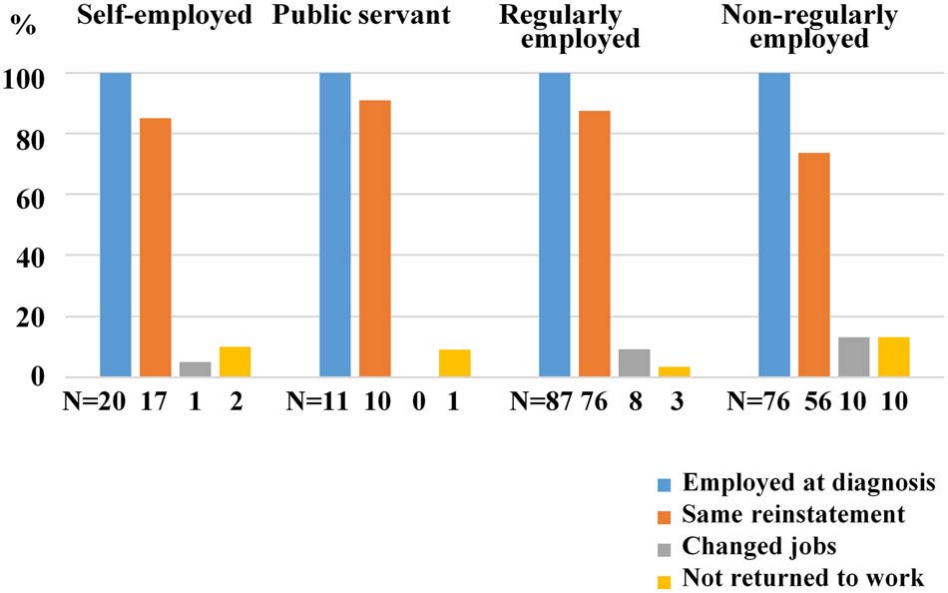
Employment status at the time of cancer diagnosis and return to work of 194 patients with gynecologic cancer.

A total of 159 patients (82.0%) returned to the same workplace, 19 (9.8%) changed jobs and 16 (8.2%) did not returning to the same workplace. Figure 1 provides the details of job changes and returning to the same workplace.

We investigated the correlations between patient characteristics and not returning to the same workplace. We used univariate and multivariable of logistic regression analyses to determine the predictive factors for not returning to the same workplace. In the univariate analysis, not being regularly employed (*P* = 0.018), short work time per day (*P* = 0.023), low personal income (*P* = 0.004), not taking sick leave (*P* < 0.001), advanced cancer stage (*P* = 0.018) and long treatment time (*P* = 0.032) were significantly associated with not returning to the same workplace. In multivariable analysis, not being regularly employed (*P* = 0.049), not taking sick leave (*P* < 0.001) and advanced cancer stage (*P* = 0.041) were significantly associated with not returning to the same workplace. Interestingly, not taking sick leave was strongly associated with not returning to the same workplace (Table 2).

**Table 2:** Logistic regression analysis for not returning to the same workplace

		Univariate analysis			Multivariable analysis	
Period	Odd ratio	95% CI	*P*-value	Odd ratio	95% CI	*P*-value
Employment pattern (non-regular employment)	2.452	1.165–5.162	0.018^*^	2.373	1.005–5.602	0.049^*^
Work hours per day (≤5)	2.451	1.129–5.321	0.023^*^	1.059	0.390–2.875	0.91
Personal income (<1 030 000 yen)	3.096	1.440–6.657	0.004^*^	1.314	0.481–3.589	0.595
Not taking sick leave	5.292	2.441–11.470	<0.001^*^	5.418	2.228–13.177	<0.001^*^
Stage (advanced stage)	2.669	1.182–6.028	0.018^*^	2.67	1.041–6.846	0.041^*^
Period of treatment (≥3 months)	2.245	1.070–4.711	0.032^*^	2.027	0.864–4.759	0.104

Abbreviation: CI, confidence interval.

^*^ *P* < 0.05.

Furthermore, we investigated the correlations between patient characteristics and not taking sick leave. In the univariate analysis, few work hours per day (*P* < 0.001), small number of people in the workplace (*P* < 0.001), low personal income (*P* < 0.001) and low household income (*P* = 0.013) were significantly associated with not taking sick leave. In multivariable analysis, low personal income (*P* = 0.030) was significantly associated with not taking sick leave. Interestingly, low personal income was significantly associated with not taking sick leave (Table 3).

**Table 3:** Logistic regression analysis for not taking sick leave

		Univariate analysis			Multivariable analysis	
Period	Odd ratio	95% CI	*P*-value	Odd ratio	95% CI	P-value
Work hours per day (≤5)	4.791	2.370–9.683	<0.001^*^	2.179	0.899–5.281	0.085
Workplace number of people (≤5)	3.651	1.835–7.262	<0.001^*^	2.052	0.948–4.441	0.068
Personal income (<1 030 000 yen)	5.588	2.765–11.295	<0.001^*^	2.679	1.103–6.506	0.030^*^
Household income (<3 000 000 yen)	2.478	1.211–5.071	0.013^*^	1.635	0.728–3.674	0.234

^*^ *P* < 0.05.

## Discussion

Gynecologic cancers are one of the most common types of malignancies in working-age women. Survival rates are rising year by year, owing to the improvements in cancer treatment methods, and the number of cancer survivors is also on the rise. The revision of the Cancer Control Act in Japan in 2016 enabled patients to receive not only an appropriate cancer treatment but also medical and emotional support, simplifying their reintegration into society. Employers are obliged to consider the continued employment of patients undergoing cancer treatment and to cooperate with the cancer control measures taken by the government (8). To date, no research on the change in status of returning to the same workplace after such measures were implemented have been published. Hence, we compared changes in the status of returning to the same workplace between 2015 (7) and 2023 (this study) at our department. In 2023, 82.0% of respondents had returning to the same workplace, 9.8% had changed jobs and 8.2% had not returning to the same workplace. Compared with the survey conducted 7 years before, the frequency of returning to the same workplace had increased, and the frequency of changing jobs and not returning to the same workplace had decreased. Therefore, it seems that the revision of the Cancer Control Act contributed to an improvement in the number of women with gynecologic cancer returning to the same workplace. Interestingly, in our previous study, non-regular employment was the variable most likely to negatively affect a returning to the same workplace (7). By contrast, in this study, not taking sick leave was significantly associated with not returning to the same workplace.

Not taking sick leave was positively related to short work time per day, small number of people in the workplace, low personal income and low household income. Interestingly, low personal income was significantly associated with not taking sick leave. However, in Japan, no laws protect employees who are not able to work owing to sickness. The maximum duration of sick leave allowed varies among companies. Non-regular employees are not commonly awarded sick leave. In fact, many small- and medium-sized enterprises in Japan do not have an established sickness insurance system (8). Individuals with cancer who are self-employed, non-regularly employed or employed by small- and medium-sized enterprises may not be able to take sick leave owing to economic circumstances. Sick leave is more common in European countries, and in certain countries, insurance covers wages during sick leave (11).

We acknowledge that our study had limitations. Most importantly, the number of patients was relatively small, and the examinations were performed at a single facility. Further prospective studies involving more patients and facilities would provide more definitive data to support the value of our results. Unfortunately, the questionnaire covered only the status of returning to work and taking sick leave, not the reasons.

In conclusion, compared with our previous survey, conducted 7 years before this one, the proportion of patients who returning to the same workplace increased from 71.3 to 82.0%. Among the 18% of women with gynecologic cancer who did not returning to the same workplace, a failure to take sick leave might have been a major contributing factor. Social support and institutions should be established to assist such women to returning to the same workplace. Therefore, we suggest that steps be taken to formally introduce a sick leave system over and above the paid leave system in Japan.
